# An Examination of Women Experiencing Obstetric Complications Requiring Emergency Care: Perceptions and Sociocultural Consequences of Caesarean Sections in Bangladesh

**DOI:** 10.3329/jhpn.v30i2.11309

**Published:** 2012-06

**Authors:** Rasheda Khan, Lauren S. Blum, Marzia Sultana, Sayeda Bilkis, Marge Koblinsky

**Affiliations:** ^1^icddr,b, GPO Box 128, Dhaka 1000, Bangladesh; ^2^John Snow, Inc., Arlington, VA, USA

**Keywords:** Caesarean section, Childbirth, Economic burden, Obstetric complications, Perceptions, Qualitative studies, Bangladesh

## Abstract

Little is known about the physical and socioeconomic postpartum consequences of women who experience obstetric complications and require emergency obstetric care (EmOC), particularly in resource-poor countries such as Bangladesh where historically there has been a strong cultural preference for births at home. Recent increases in the use of skilled birth attendants show socioeconomic disparities in access to emergency obstetric services, highlighting the need to examine birthing preparation and perceptions of EmOC, including caesarean sections. Twenty women who delivered at a hospital and were identified by physicians as having severe obstetric complications during delivery or immediately thereafter were selected to participate in this qualitative study. Purposive sampling was used for selecting the women. The study was carried out in Matlab, Bangladesh, during March 2008–August 2009. Data-collection methods included in-depth interviews with women and, whenever possible, their family members. The results showed that the women were poorly informed before delivery about pregnancy-related complications and medical indications for emergency care. Barriers to care-seeking at emergency obstetric facilities and acceptance of lifesaving care were related to apprehensions about the physical consequences and social stigma, resulting from hospital procedures and financial concerns. The respondents held many misconceptions about caesarean sections and distrust regarding the reason for recommending the procedure by the healthcare providers. Women who had caesarean sections incurred high costs that led to economic burdens on family members, and the blame was attributed to the woman. The postpartum health consequences reported by the women were generally left untreated. The data underscore the importance of educating women and their families about pregnancy-related complications and preparing families for the possibility of caesarean section. At the same time, the health systems need to be strengthened to ensure that all women in clinical need of lifesaving obstetric surgery access quality EmOC services rapidly and, once in a facility, can obtain a caesarean section promptly, if needed. While greater access to surgical interventions may be lifesaving, policy-makers need to institute mechanisms to discourage the over-medicalization of childbirth in a context where the use of caesarean section is rapidly rising.

## INTRODUCTION

Maternal mortality has been described as a problem that affects people living in poverty. Recent studies have illuminated higher risk for maternal death among socially- and economically-vulnerable people who have limited access to lifesaving obstetric hospital-care, including obstetric surgery (1-4). In Bangladesh which is a resource-poor country, nationwide data showed that maternal mortality has declined substantially over the past nine years, with the ratio decreasing from 322 in 2001 to 194 in 2010 (5). Some of the decline can be attributed to the increase in the number of deliveries at facilities, which more than doubled from 9% of births in 2001 to 23% in 2010. The use of caesarean sections also revealed a five-fold increase from 2.6% in 2001 to 12.2% in 2010 (5). Data from the Bangladesh Demographic and Health Surveys collected in 2000 and 2007 and more recent nationwide data showed that much of the increase in the number of births at facilities has been driven by the use of private-sector services (5-7). Hence, it is not surprising to see persisting socioeconomic inequities in facility-based deliveries (5,8).

Caesarean section is a surgical intervention designed to prevent or treat some life-threatening maternal or foetal complications. While the World Health Organization (WHO) recommends that the rates of caesarean births should range from 5% to 15% (9), there is little consensus regarding optimal rates of caesarean births (10). In middle- and high-income countries, much attention has been given to the increased use of elective caesarean sections carried out for the convenience of pregnant women. In resource-poor countries, except those in sub-Saharan Africa, there is also evidence of sharp increases in rates of caesarean sections as childbirth is increasingly occurring in health facilities with skilled attendants, particularly in urban settings and among wealthier people (3). In these settings, data relating to medical indications for caesarean section are lacking, thereby increasing the challenge of determining the percentage of cases that warranted caesarean surgery. However, in the 1990s, the lack of decline in maternal mortality ratios that coincided with a sharp increase in caesarean sections in resource-poor settings suggests that women delivering by caesarean section were not those in the greatest need (10). Given the high costs involved, socioeconomic disparities in access to caesarean-section procedures persist (3), and there continues to be concern that poor women who need a caesarean section may not get it, and other women who can afford to pay for a caesarean section but do not experience life-threatening complications get a caesarean section unnecessarily.

Social scientists have examined costs involved in emergency obstetric care (EmOC) and the subsequent economic burden on women and their families (11-13). However, little is known about beliefs regarding caesarean sections in cultural frameworks where deliveries at home are preferred and how the economic burden resulting from a caesarean section impacts on broader cultural and social consequences to women and their families, particularly those living in poor households located in low-income, rural settings. With the recent increase in the number of caesarean births in Bangladesh, research is needed to examine the understanding of and demand for caesarean sections and the social and psychological consequences on women undergoing a caesarean section.

To address this gap in knowledge, qualitative research was carried out among women who experienced severe obstetric complications to understand the perceptions of caesarean section and how having obstetric surgery affects women and their families. The study examined women who did and did not have a caesarean section for their birthing preparation, delivery-related experiences, and subsequent physical, social and economic consequences for the woman and her family members up to 10 weeks after delivery.

## MATERIALS AND METHODS

### Study site

The study was carried out during March 2008–August 2009 in Matlab, Bangladesh. Matlab is a rural area 45 km southeast from the capital Dhaka, in a deltaic region of the country. In Matlab, a Health and Demographic Surveillance System has been maintained by icddr,b since 1966; icddr,b maintains a hospital and four subcentres that provide free child- and maternal care (antenatal, intrapartum, normal and basic emergency and postpartum care, and family planning), along with general health services, for a population of 110,000 in Matlab. The paramedics of the four subcentres assist in normal deliveries and refer women either to the Matlab Hospital of icddr,b which offers basic EmOC that can be handled by physicians or to local hospitals that provide comprehensive EmOC. Community Health Workers (CHWs) visit all families living in the icddr,b intervention area once every two months to identify pregnancies and capture vital statistics. They provide antenatal care to pregnant women and postpartum care and also general healthcare to families during bi-monthly household visits. Other local health services facilities in the area include one government hospital in the district town, two government facilities with EmOC in the district town, over 20 private facilities offering EmOC, and an array of informal healthcare providers. At the time the study was carried out, 66% of deliveries took place in facilities (14).

### Study sample and participants

Within 8-10 weeks after delivery, in-depth interviews were conducted with 20 women who delivered in a hospital and who were identified by physicians as having severe obstetric complications. While the postnatal period extends up to six weeks after delivery, a longer timeframe was needed to locate the respondents. Purposive sampling was used for identifying women who had suffered a range of severe complications from a list of samples provided by the physicians who participated in a larger quantitative study examining the community-based prevalence of maternal morbidities (15). The complicated cases included women with haemorrhage (n=6), eclampsia (n=2), severe anaemia (n=4), and dystocia (n=8), all of whom had delivered in one of the local hospitals offering basic or comprehensive EmOC. Informal discussions were also carried out with family members of those women who lost consciousness or could not relay entirely what had happened during events relating to care-seeking and costs associated with the delivery. When available, husbands were interviewed for complete details about the childbirth. Five husbands were included in the study.

### Data-collection procedures

Seven researchers with training in anthropology conducted the interviews which were carried out in Bangla. The researchers followed guidelines that examined various research themes and topics, including birth planning and preparation, ANC, care-seeking behaviours during labour and delivery, perceptions of emergency-care procedures, particularly caesarean sections, support provided by family members before and after the delivery, physical and psychological consequences associated with delivery procedures, and the economic impact of EmOC on families. Interviews lasted, on an average, for two hours. Efforts were made to carry out the interviews in a private location, either inside the household or in the family-yard. All interviews were tape-recorded and later transcribed and translated into English. Follow-up visits were made 2-3 months later to interview half of the respondents to capture information that was missing during the first interview. While the contents of the follow-up interviews varied according to the initial data collected, interviews often involved gathering information relating to the sequence of care-seeking, the duration of the different stages of treatment, and the costs involved in obtaining healthcare.

### Analysis of data

A code-list was developed based on the main research themes outlined above. ATLAS.ti, a text-organizing software, was used for coding the interview transcripts. Once the data were coded, content analysis was used for identifying and comparing the trends of key concepts in the coded data according to the type of respondent. Data triangulation was employed to identify those concepts that could be validated through a combination of data sources, such as multiple persons interviewed. The quantitative data were analyzed using SPSS 11.5. Factor analysis of household assets conducted by icddr,b on all the households in Matlab was employed to assess wealth ranking. Monetary figures are primarily presented in local currency—the Bangladeshi Taka (Tk). During the study period, Tk 69 was equivalent to approximately US$ 1.00.

### Ethical aspects

Before participating in the study, verbal informed consent was obtained from all the respondents. The Ethical Review Committee of icddr,b approved the study.

## RESULTS

### Background information

The women interviewed were, on average, in their mid-20s (Table 1). Over half of the respondents were educated beyond primary school, and three had more than 10 years of education. None of the women was involved in income-generating activities. Most women were living in their in-laws' home at the time of the last pregnancy and delivery. Over one-third of the women were first-time mothers. Husbands were, on average, in their early thirties, and most of them received education beyond primary school. More than half held relatively high-income occupations involving businesses or overseas jobs. The household wealth indices showed that more than half of the women came from households in the two highest quintiles.

### Antenatal care and birth planning

Almost three-fourths of the women had 3-4 ANC visits. During these visits, they were counselled on nutritional requirements and the importance of taking adequate rest during pregnancy and were advised to attend postpartum check-ups for the mothers and the babies. Although the health workers recommended that the women deliver at a health facility with skilled attendants, the possibility of a caesarean section, if complications occurred, was not discussed even with the two mothers who had previously undergone caesarean sections. Consequently, saving of money for such emergency was also not discussed.

**Table 1.** Background information of women having complicated deliveries

Variable	Complicated deliveries (n=20)
Average age (years) of woman	24.9
Education of women	
No education	2
Grade 1 to 5	6
Grade 6 to 10	9
Beyond Grade 10	3
Parity of the last pregnancy	
First	7
Second	8
Third	4
Fourth	1
Fifth or more	0
Where women resided during pregnancy	
In-law's home	12
Natal home	5
Nuclear home	3
Average age (years) of husbands	33.15
Education of husbands	
No education	1
Grade 1 to 5	7
Grade 6 to 10	10
Beyond Grade 10	1
Occupation of husbands	
Business	10
Carpentry	3
Work abroad	3
Services	3
Rickshaw-pulling	1
Household wealth index	
Lowest	3
Second	2
Middle	4
Fourth	6
Highest	5

Three-fourths of the women planned for a delivery at a health facility, and the remaining women had decided to give birth with a traditional birth attendant (TBA) at home. None of these women contacted the TBAs to discuss the impending birth during the pregnancy period. Six of the 20 women or their families had saved money for delivery.

### Delivery and care-seeking

The figure shows care-seeking and the place of delivery in accordance with the decisions made by the family before delivery. Six of the 20 women had vaginal deliveries, and the remaining women had caesarean sections, with four of five women who had planned on deliveries at home eventually having a caesarean section.

Three of six women who delivered vaginally suffered from haemorrhage, and three had severe anaemia. Only one of these women had originally planned on delivery at home; after she had started haemorrhaging, she sought care in two facilities before giving birth to a child at a private hospital. The other five women sought delivery care only in the basic EmOC hospital where their condition was managed. One of these women, however, bled excessively after delivery and was sent to the district hospital where she received four units of blood. While one of the haemorrhage cases was advised to have a caesarean section due to high blood pressure, she refused, claiming that it would have long-term negative effects on her health and ability to work.

Complications for the 14 caesarean-section cases included prolonged labour or a breeched baby (8 cases), high blood pressure and convulsions (2 cases), haemorrhage (3 cases), and severe anaemia (1 case). All the women who had caesarean sections sought care with at least two providers before undergoing the procedure, with all but one going to the basic EmOC hospital and subsequently being referred to an emergency facility for caesarean section. Those women who had initially planned delivery at home sought care from three or four informal and formal healthcare providers before eventually having a caesarean section. Three of the 14 caesarean sections took place in a public hospital, and the remaining 11 women had the operation in a private clinic or hospital. The medical indication for having a caesarean section was not explained to six of the 14 women.

Of the 14 women who had caesarean sections, 12 families initially refused, indicating that they were not mentally or financially prepared to spend such a large sum of money. They also expressed concern that all subsequent deliveries would have to be done by caesarean section. In addition, many families held the belief that caesarean sections were recommended so that healthcare providers could make money rather than as a lifesaving procedure for the mother and the child. Eventually, the health workers convinced the families to accept the procedure by explaining the potential life-threatening consequences to the mother or the child, negotiating the cost with the referral facility, and in some cases, providing an ambulance. One woman explained:

A caesarean section needs Tk 40,000-50,000. The economic status of my parents and husband was not strong enough to bear this cost. We, however, needed Tk 10,000 at the end. We got financial support from the hospital which paid for the operation and the hospital room. The Tk 10,000 we contributed was paid for the medicine. We managed Tk 10,000 by borrowing.

**Fig.**
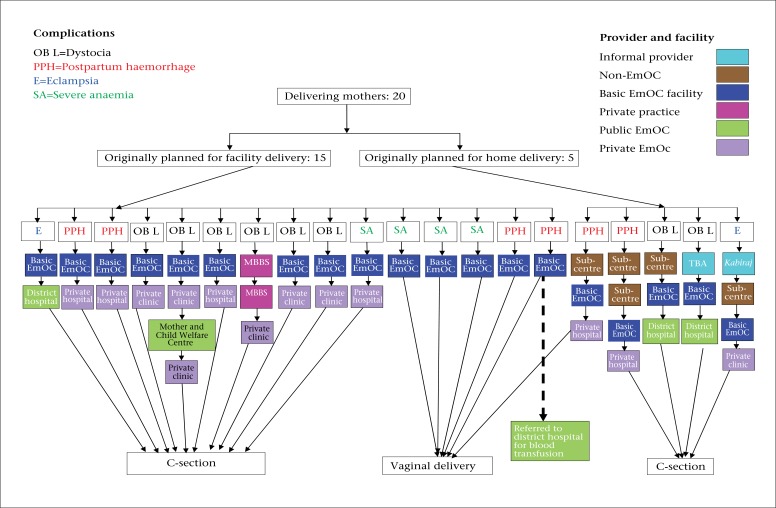
Care-seeking for and delivery by women who suffered from severe complications

The remaining two of the 14 women had previously had caesarean section and assumed that they would have to go for the procedure again during this pregnancy.

### Health consequences

Table 2 shows that women with severe complications cited numerous health consequences, with the most common relating to problems with their breasts, weakness, bodyache, bleeding for more than a month, and fever. Sleep deprivation, pain or fear of pain during urination and defaecation, and changes in body-colour were also commonly mentioned. No treatment was sought for the most commonly-reported conditions, including weakness, bodyache, or problems with breasts; many of the other problems listed were believed to be resolved over time with medicines provided by the hospital.

Only minor differences in health consequences and treatment-seeking were noted between the caesarean section and the vaginal delivery cases at the time of the interviews. Conditions unique to caesarean cases included pain and infection in the stitched areas. The two women who had had caesarean sections during previous births also expressed many other concerns relating to their future, such as developing infections in the stitched area, bearing children who would be more susceptible to disease and require extra care, and the fear that they would not be able to have more than two children.

**Table 2.** Health consequences reported after childbirth by women having complicated deliveries

Health consequence	Complicated deliveries (n=20)
Breast problems, including pain, engorgement, and overflow of milk	22*
Weakness	18
Bodyache/pain (e.g. waist, back, shoulder, leg, chest, head, and burning)	16
Prolonged bleeding up to 45 days	14
Fever	11
Pain in the stitched area	10
Deprivation of sleep	9
Problems with skin (e.g. colour change, spots or blotches, and cracked skin)	8
Fear of/or pain in urination and defaecation	8
Mental irritation	7
Excessive bleeding	4
Infection in the stitched area	3
Weight loss	3
White discharge	3
Pain/irritation in the birth canal	2
Lower abdomen pain	2
Breathing problems	2
Heaviness at the mouth of vagina	1
Pain in urinating	1
High blood pressure	1
Hernia	1

*Some women mentioned more than one problem relating to their breasts

### Daily activities and support from family members

Women continued doing regular chores during pregnancy and up to the time of delivery while heavy chores, such as carrying water, washing clothes, and lifting heavy items, were restricted during the last trimester. Of the 12 women who were with their in-laws before and after childbirth, nine resumed normal chores 8-10 days after delivery, avoiding heavy chores for up to one and a half months. These women received support from their in-laws who provided them with nutritious foods and assisted in caring for the newborn and older children. The other three women, all of whom had caesarean sections, did not receive aid from their in-laws during the postpartum period, with two explaining that the in-laws were angry over having spent so much money on the delivery and the third woman indicating that there was a family dispute regarding dowry.

The five women who were in their natal families were not permitted to carry out even normal chores up to one month postpartum. The family members also provided other support, including accompanying women for visits to the hospital and performing household chores for the new mother even when she had returned to the in-laws' residence. The remaining three women who resided at nuclear homes received little assistance, having to resume all household chores just a few days after delivery.

No major differences regarding ability to carry out work were identified between women who had or did not have caesarean sections. The majority of the women mentioned that following delivery, health consequences such as weakness delayed their ability to care for themselves and perform critical chores adequately. Specifically, the women mentioned that they were unable to feed and clean their babies and give emotional comfort as required, give adequate attention to the preparation of food for older children and oversee their educational needs, or carry out primary family chores, such as preparing food or washing clothes. Discomfort linked to backpain and pain in the stitched area for those women who had caesarean sections, particularly when sitting, was frequently mentioned as a problem that interfered with cooking.

Women who experienced caesarean section expressed grave concerns that the procedure would severely impact on their productivity in the future, particularly their ability to perform heavy chores, such as carrying jugs of water, washing clothes, lifting heavy objects, especially crops during the harvest season, or carrying children. All these women were worried about who would assist with these chores and whether they would be able to care for their children and other family members as they did before the caesarean section, with many believing that their changed physical capabilities might jeopardize their marital status and family life. One woman stated:

No one in my family has had a caesarean operation. How unlucky I am! What will be my physical condition in the future? My life has no meaning since I had the operation. Earlier, I could do any hard work. Now I will be unable to do heavy chores as I did before; I will have to live my life like a patient.… People do not take the caesarean operation easily. They say Allah proved that she is not a good person by cutting her abdomen.

### Family economy

Delivery-related costs are presented in Table 3. Of the five women who delivered vaginally in the basic EmOC hospital, three spent Tk 500-1,500, and the other two who had a longer hospital stay paid Tk 10,000-14,999, with one of these women also requiring a blood transfusion in the district hospital. The other woman who delivered vaginally in a private hospital paid Tk 6,000. Household wealth index information showed that one family came from the poorer socioeconomic strata.

**Table 3.** Costs incurred for women experiencing vaginal or caesarean-section deliveries during an obstetric complication

Cost (Tk)*	Complicated deliveries	
	Vaginal deliveries (n=6)	Caesarean-section deliveries (n=14)
Up to 4,999	3	0
5,000-9,999	1	0
10,000-14,999	2	5
15,000-19,999	0	4
20,000 or more	0	5

*At the time of the study, Tk 69 was equivalent to US$ 1

Of the 14 caesarean-section cases, five women spent Tk 10,000-14,999, with four delivering in a private and one in a public facility. Four women spent Tk 15,000-19,999, all of whom went to a private facility, and the remaining five families spent Tk 20,000-35,000, with three delivering in a private and two in a public facility. Payment of larger sums of money generally reflected longer periods in the hospital postpartum, with three women incurring large costs to treat an infection in the stitched area that required medical care. Twice as many women who had caesarean sections came from families of the wealthier socioeconomic strata compared to those from the poorer socioeconomic groups.

Of the six families who had saved money for delivery, three (two paying for vaginal deliveries and one for a caesarean section) had put aside enough money to cover expenses. Savings of the other three families, all of whom had to pay for a caesarean section, were seriously insufficient to cover healthcare costs. Overall, nine of the 14 families of women who had caesarean sections were forced to borrow money from microcredit institutions or relatives. Some of these families faced serious problems in repaying the loan, which included interest. One woman explained:

It became a huge burden for my husband. It would have been good if we could have avoided the caesarean section and I had delivered normally.

These families were either counting on the next harvest season or trying to reduce family costs to repay the loan. Women also indicated that their husbands worked harder and for longer hours to increase earnings. In three cases, natal families bore the entire costs because the in-laws were either unable or refused to contribute. In these instances, women stated that their fathers either sold family-land or took loans with interest, creating a severe impact on the economic status of the families, which were already poor. In one case, a serious conflict between the natal and the in-law families ensued over the cost of caesarean section; the in-laws refused to contribute to expenses and insisted that the natal family sell the woman's gold ornament. Of the remaining five families, one husband sent money from abroad, one couple had saved money in case an emergency arose during delivery, and in three cases, both natal and husband's families shared the expenses.

Women experiencing a caesarean section were generally distraught about the cost involved in the delivery, which represented a tremendous economic burden to the family, with many regretting that they had not saved money during the pregnancy. These women indicated that they could not enjoy the arrival of the baby because they were too preoccupied with the costs incurred. One woman explained:

I was happy with the pregnancy … however, I was confused about what to feel just after the caesarean section. I was thinking about the cost. I was not in a physical and mental condition to feel happy as I would have been if I had delivered the child normally.

Four women were blamed by their in-laws for incurring such huge expenses for the caesarean section, with one of them also developing an infection and causing further economic hardship for the family. The resulting burden that the women were accused of causing induced in them both shame and guilt, with several indicating that they were responsible and deserved the blame. One woman said:

I do not say anything to my mother-in-law when she blames me. I have to take this since I am guilty, and I cost so much money.

One of these women claimed that her situation would have been worse if she had delivered a daughter, stating:

I was lucky that it was a boy. I already had a daughter. If this one were a girl, it would not have been possible for me to have a third child quickly in hope of having a boy. The reason is that I would have to save money first for a caesarean section and then get pregnant.… My deliveries are a matter of cost. So, when I delivered a boy, everybody was happy.

Many women expressed concerns about the next pregnancy which they believed would also have to involve a caesarean section. As a result, these women indicated that they would not be able to have another child as they wished, doubting that they could save the money required, with many expressing resentment and frustration for their problems. One mother said:

My mother and sisters did not need a caesarean section. They did not need to spend any money for delivery. Why did I need to do that? I will not be able to take more than one or two child(ren). This time everyone helped me financially but who is going to help me next time? My father provided support this time; will he be alive during my next pregnancy?

## DISCUSSION

This in-depth study examining the social and economic consequences for women who had recently delivered with severe complications revealed three major barriers that influence care-seeking to obtain EmOC and acceptance of surgical procedures. First, ANC consultations provide little information regarding obstetric complications and medical indications for caesarean section or where to go for emergency care. Second, women have misconceptions and concerns about caesarean section and distrust health workers regarding the reasons they recommend the procedure. And third, women who had a caesarean section incurred enormous costs that often led to economic burdens on family members and blame attributed to the woman. These findings highlight the need to inform families about caesarean section and encourage them to prepare in advance in the case of delivery-related complications.

The most obvious time to convey information about delivery is during ANC. Although ANC visit attendance was relatively high, the information shared during consultations focused on habitual care for women during pregnancy, with minimal information given about childbirth and unpredictable maternal complications or the importance of setting aside savings. Medical indications for caesarean section and the reasons for needing the procedure were not discussed even with women who had previously experienced a caesarean section. These findings coincide with recent nationwide data showing that only one-third of women receive advice on danger signs during ANC visits (5), even though the visits provide an ideal opportunity to raise women's awareness about pregnancy-related complications that may require a caesarean section as a lifesaving measure and encourage families to prepare mentally and financially in case an unexpected emergency occurs.

Three-fourths of the women with complications had planned delivery at a facility, thus reflecting the recent shift in the greater use of skilled birth attendants in Matlab and Bangladesh in general. Yet, women suffering severe complications faced difficulties in accessing facilities that could address their medical needs, highlighting the need to inform women during ANC about where to obtain EmOC. Basic EmOC facilities should be able to manage the haemorrhage cases if they come early, and in the case of five of the eight women with haemorrhage or severe anaemia who went directly to the basic EmOC hospital, they appeared to have been managed adequately at that site; the other three women were referred to private hospitals and had caesarean sections. Most women with other complications (e.g. dystocia, eclampsia) ultimately went to private facilities where the supplies and staff needed to manage complications are typically perceived as more accessible. Given that there are 10 times more private than public facilities, this perception is accurate. Yet, 15 women went to two to four facilities before obtaining appropriate treatment, again showing the need to inform families about which health facilities provide care for what problems.

A major misconception relating to having a caesarean section was that the woman would no longer be capable of performing heavy work and must rely on other family members for assistance with household chores. In Bangladeshi society, where the social framework enforces the belief that the primary roles of female involve childbearing, taking care of family members, and carrying out responsibilities to ensure maintenance of the household, failing to meet these critical social responsibilities renders women vulnerable to ridicule and rejection and even marital dissolution (16,17). Another assumption was that subsequent deliveries would also have to be through caesarean section, thus raising uncertainty about whether these women could afford to bear additional children, once again potentially undermining their status as wives and women. Results of other studies in Bangladesh and elsewhere also identified negative connotations associated with caesarean section, including fear of death, fear of infertility, concerns about resultant restrictions with regard to performing household work, and fears about future childbearing (18-20), and a strong preference for vaginal delivery (21-26).

A compelling reason for deterring or delaying the use of health services or refusing a caesarean section is the cost involved (18,27,28), which is beyond the means of poor families and far greater than expenses incurred by the study women who experienced severe complications but delivered vaginally, or for those with normal deliveries, particularly those that occur at home (29). The majority of the families of women who had a caesarean section spent over Tk 14,999 (US$ 217), approximately one-third of the annual GDP per capita in Bangladesh (30). The most costly cases spent up to Tk 35,000 (US$ 507) due to lengthy stays in the hospital and/or subsequent infection. Payment for over half of the women with caesarean sections made it necessary for families to borrow money and incur debt, thus putting major strains on the household economy and requiring some families to enforce severe restrictions on spending that could impact the well-being of other family members. This was true, even though twice as many women who had caesarean sections came from families in the higher wealth strata compared to those coming from the poorer socioeconomic groups. In comparison, the families of women who delivered vaginally did not experience such economic hardship. Since the interviews were carried out up to 10 weeks postpartum, we could not assess the longer-term economic consequences for families of women who had experienced caesarean sections. The poorer families, however, anticipated that they would not be able to pay the debt over a prolonged period, probably up to the time of the subsequent harvest. The fact that many repaid their debts within six months after delivery is discussed in a paper by Powell-Jackson *et al.* (31). Other research has also described the economic burden poor families in Bangladesh confront in paying for emergency obstetric services (27). Relying on future assets can spiral households into a vicious cycle of debt and impoverish families or force them into deeper poverty as has been shown for some catastrophic health events, including obstetric emergencies (11,12,32,33).

Disputes over payment for expenses for the caesarean section also ensued, causing rifts between the woman's in-laws and the natal families. Women were frequently blamed for undergoing the procedure and incurring expenses that caused economic burden on households; perceptions associated with the physical consequences of caesarean section and the economic implications made women feel culpable and believe that the blame was, therefore, justified, with some suggesting that the delivery had negative effects on their lives. Families also expressed scepticism about the health provider's reason for recommending a caesarean section, with many believing that they were prescribing the procedure for their economic gains rather than medical reasons. Results of another study in Bangladesh also showed distrust in the recommendations of doctors due to perceptions that caesarean sections were being administered for personal gain, a conviction which served to dissuade women from visiting a health facility out of fear that a caesarean section might be unnecessarily performed and high costs incurred (28). Overall, the negative perceptions identified in this study about the physical and social consequences associated with caesarean section influenced many women and their families to refuse the procedure initially, even when they suffered life-threatening complications. In these cases, counselling and support by the health worker in the form of transport and a negotiated reduction in the cost often served as a catalyst for families to accept surgical intervention. This information underscores the importance of appropriate and timely medical advice to convince families about the urgency of the situation and the medical indication for surgical birth as a lifesaving option and strong referral systems that facilitate efficient and affordable care for those in need.

The global trends in caesarean section, in fact, suggest that the procedure is often performed when there is no medical indication of need and so done for the convenience of the healthcare provider or the woman or for the economic benefit of practitioners (10), confirming family concerns. The high fees for caesarean sections, which, on an average, were reported to be Tk 19,428 (US$ 282) in our study can, indeed, garner the healthcare provider large sums of money and serve as a strong financial incentive to recommend the procedure. Nationwide data recently collected in Bangladesh showed a sharp rise in the use of caesarean section which increased by five-fold from 2.6% in 2001 to 12.2% in 2010, with two-thirds being carried out in private facilities, which in itself has implications regarding insufficient access for poor populations (5). In the present study, 14 of the 20 women with severe complications had caesarean sections. Moreover, a higher percentage of caesarean-section cases belonged to wealthier families compared to women who delivered vaginally, coinciding with nationwide data showing persisting socioeconomic inequities in facility-based and caesarean-section deliveries in Bangladesh (5,8).

While the rates are still below the 15% WHO-set maximum threshold, disparities between the rich and the poor suggest a trend to perform caesarean sections unnecessarily. To reverse this pattern, policy-makers should take measures to restrict unnecessary procedure, while at the same time ensuring that all women with life-threatening complications obtain surgical intervention as needed. In Bangladesh, a voucher scheme is now being implemented to ensure that women have access to delivery care, including caesarean sections, when needed. Just how effective this new effort will be in addressing the actual need, while curbing the abuse of costly services, is still unknown (31).

Women mentioned a constellation of health consequences of postpartum complications. Most of these problems were considered normal for a mother who recently delivered and, therefore, treatment was rarely sought. Data from Burkina Faso also found that medical needs of women suffering from health consequences postpartum were not adequately met (34). The differences uncovered in the present study relating to those consequences were directly linked to the caesarean-section incision, which in several cases caused infection and necessitated additional medical attention and costs, which warrants further investigation on the quality of care provided. While we were unable to confirm the reliability of these or the other self-reported morbidities, the extensive list in Table 2 represents both psychosocial and physical consequences, some potentially severe in nature, that may reflect women's roles and pressures in the social environment postpartum. While these conditions should not be ignored, local interpretations of illness, cultural perspectives of reproductive health, and women's needs are formulated in a particular social context that guides the perceived requirement for medical care (34,35).

The respondents reported difficulties fulfilling some of their basic household responsibilities, including caring for the newborn, highlighting the need to give them adequate rest and time for recovery postpartum. Women who were in their natal homes postpartum received far more physical support than those who remained with their in-laws, regardless of the mode of delivery. This is likely the reason that Bangladeshi women traditionally prefer to spend the last trimester and give birth in their natal households, where family members restrict them from performing chores and allow women postpartum to spend more time with the newborn. As the number of nuclear families increases, this tradition appears to be gradually changing, thus forcing more women to engage immediately in work postpartum and depriving new mothers of this critical recuperation period and more intensive time with the newborn.

### Conclusions

The findings of this study underline the importance of educating women and families during pregnancy about pregnancy-related complications and preparing for the possibility of caesarean section. Such efforts can take place during improved antenatal consultations, which at present appear to provide only limited information about danger signs, complications, and where to go for appropriate services. Raising awareness about caesarean section should help dispel fears about going to health facilities and increase the proportion of women willing to deliver with skilled birth attendants, which continues to be low in Bangladesh at 27%, and decrease care-seeking from alternative, often unqualified and traditional practitioners who may use harmful practices. At the same time, during labour and delivery requiring surgical intervention, medical professionals should elicit women's childbirth expectations and beliefs and concerns about obstetrical procedures and explain the medical intervention so that it is mutually understood as a procedure suitable to the care provider and patient. Such efforts will establish trust and allow families to make informed and quicker decisions about whether or not to accept the procedure and so should improve obstetrical satisfaction and outcomes.

Within a very short timeframe, Bangladesh has nearly reached the maximum threshold of 15% of births delivered through caesarean section, as stated by the WHO. Paradoxically, this rapid change in the rate of caesarean section is taking place in a context where cultural norms and traditional birthing practices have historically placed Bangladesh among the countries with the lowest rates of skilled birth attendance. While greater access to surgical interventions may be linked to recent reductions in maternal mortality, the sharp increase that has occurred over the past decade also raises concerns that the country will follow the trends established in other middle- and high-income countries where childbirth is highly medicalized, and the use of caesarean section continues to rise despite lack of evidence in improved maternal and perinatal mortality and morbidity (26). At the same time, the negative perceptions towards caesarean sections identified through this study can influence misinformed decisions about birth attendance and mode of delivery and put women experiencing life-threatening complications at risk. Policy-makers, thus, need to develop protocols regarding appropriate caesarean-section practices and institute mechanisms to discourage the over-medicalization of childbirth and commercialization of caesarean sections, and, at the same time, ensure that those women in clinical need of lifesaving obstetric surgery have access to obtaining a caesarean section and understand the need for it. Finally, additional research is needed to examine perceptions of caesarean section from the standpoint of women from a range of socioeconomic backgrounds and to identify the household factors favouring surgical interventions and their impact on birth outcomes.

## ACKNOWLEDGEMENTS

The study was funded by the United States Agency for International Development (USAID), Washington, DC. The authors acknowledge the physicians who helped identify women who met the criteria to participate in the study. The authors also thank the CHWs of the Matlab Health and Demographic Surveillance System area, who assisted in locating the homes of informants. The authors are grateful to the study respondents who gave their valuable time to participate in the study.
